# Dr. George W. Gorrill

**Published:** 1918-12

**Authors:** 


**Dr. George W. Gorrill,** Buffalo 1900, Supt. of the Buffalo State Hospital, died suddenly Oct. 27, aged 41. He was born in Ontario, served as intern at the Sisters' Hospital, joined the staff of the State Hospital Jan. 9, 1902, was made first assistant physician in March 1911 and succeeded Dr. Hurd as Supt., July 29, 1918.

